# THE RELATIONSHIP BETWEEN LIVING ALONE AND STRESS FROM A LIFE COURSE PERSPECTIVE

**DOI:** 10.1093/geroni/igae098.3752

**Published:** 2024-12-31

**Authors:** Junyub Lim, Ross Andel, Katerina Sheardova, Jan Pavlik, Jana Amlerova, Jakub Hort

**Affiliations:** Arizona State University, Phoenix, Arizona, United States; Arizona State University, Phoenix, Arizona, United States; International Clinical Research Center, St. Anne’s University Hospital, Brno, Jihomoravsky kraj, Czech Republic; AlzheimerChain Foundation, Prague, Hlavni mesto Praha, Czech Republic; Second Faculty of Medicine, Motol Epilepsy Center, Charles University, Prague, Czechia, Prague, Hlavni mesto Praha, Czech Republic; Second Faculty of Medicine, Charles University, Motol University Hospital, Prague, Czechia, Prague, Hlavni mesto Praha, Czech Republic

## Abstract

**Background/objectives:**

Social isolation may affect psychological well-being, but understanding this link across adulthood is limited. We examined living alone in relation to dealing with daily stress among younger, middle-aged, and older adults.

**Methods:**

Data from Terrapino, a new mobile application designed to support cognitive health, were utilized. Psychological well-being was assessed with a 10-item index of dealing with daily stress over the past month (get angry, feel like things are piling up, feel like being on top of things, etc.) reported as never/sometimes/often/very often. Demographic, health, and lifestyle information was also collected.

**Results:**

Complete data were available for 6,154 users who gave consent to allow reports of aggregate data (mean=57.2±14.4, range 18-103 years, 73% were female). Users were categorized as young (18-30 years, n=331), middle-aged (31-60 years, n=3,058), and older (61+ years, n=2,765) adults, of whom 45% (52% male/42% female), 20% (17% male/21% female), and 32% (15% male/39% female) young, middle-aged, and older adults, respectively, lived alone. Controlling for sex, education, and comorbidity, young adults who lived alone had poorer ability to deal with daily stress (Estimate=0.22, p<.05), no differences existed among middle-aged adults (p>.05) and better ability to deal with daily stress emerged among older adults living alone (Estimate=-0.11, p<.01).

**Discussion:**

Using data from a freely available mobile application, young users who lived alone dealt with daily stress relatively poorly, whereas their older counterparts showed opposite trends regardless of comorbidity. This study sets the stage for research into mobile application use to improve well-being of older adults living alone.

